# Defense-related proteins involved in sugarcane responses to biotic stress

**DOI:** 10.1590/1678-4685-GMB-2016-0057

**Published:** 2017-02-20

**Authors:** Thais P. Souza, Renata O. Dias, Marcio C. Silva-Filho

**Affiliations:** 1 Departamento de Genética, Escola Superior de Agricultura Luiz de Queiroz, Universidade de São Paulo, Piracicaba, SP, Brazil

**Keywords:** Defense-related protein, PR-protein, biotic stress, *Saccharum* spp

## Abstract

Sugarcane is one of the most important agricultural crops in the world. However, pathogen infection and herbivore attack cause constant losses in yield. Plants respond to pathogen infection by inducing the expression of several protein types, such as glucanases, chitinases, thaumatins, peptidase inhibitors, defensins, catalases and glycoproteins. Proteins induced by pathogenesis are directly or indirectly involved in plant defense, leading to pathogen death or inducing other plant defense responses. Several of these proteins are induced in sugarcane by different pathogens or insects and have antifungal or insecticidal activity. In this review, defense-related proteins in sugarcane are described, with their putative mechanisms of action, pathogen targets and biotechnological perspectives.

## Introduction

Sugarcane (*Saccharum* spp. hybrids) is the primary source of sugar and renewable biofuel energy worldwide. With high carbohydrate content, favorable energy input/output ratio and high biomass production capacity, sugarcane is one of the best options to generate biofuel (Hoang *et al.*, 2015). The growing global demand for energy has led to increased interest in the development of new sugarcane cultivars with high productivity for use as bioenergy feedstock (Hoang *et al*., 2015).

Sugarcane production is constantly challenged by different abiotic and biotic stresses (Long and Hensley, 1972; Azevedo *et al.*, 2011). Biotic stresses include, among others, fungal infection by species such as *Colletotrichum falcatum* (red rot disease), *Sporisorium scitamineum* (sugarcane smut), *Fusarium* spp. and *Ceratocystis paradoxa* (pineapple disease), bacterial diseases such as red stripe (*Acidovorax avenae*), leaf scald (*Xanthomonas Albileneans*) and sugarcane grassy shoot disease (Phytoplasma), virus infection by species such as sugarcane mosaic virus (ScMV) and yellow leaf virus (ScYLV), and insect attack by species such as *Diatraea saccharalis* (sugarcane stem borer), *Eldana saccharina* (African sugarcane stalkborer) and *Sphenophorus levis* (sugarcane weevil).

Plants respond to biotic stress by constitutive or inducible defense mechanisms. Many defense related (DR) proteins have been identified in different plants after infection by fungi, oomycetes, bacteria and viruses or attack by insects and nematodes (Van Loon, 1999; Van Loon *et al*., 2006).

Plant microbe-induced proteins and their homologs are usually collectively called as “pathogenesis-related” (PR) proteins. However, this concept has been revised to include only proteins induced by pathogens and which are mostly not detectable in their absence (Van Loon *et al.*, 2006). In plant defense responses, PRs affect pathogen or herbivore development (Ryan, 1990; Bohlmann and Broekaert, 1994; Broekaert *et al.*, 1995) or stimulate plant defensive barriers (Van Loon *et al.*, 2006).

Other proteins are also involved in plant defense, such as NBS-LRR proteins, which recognize a wide variety of pathogens and insects (Li *et al.*, 2015), glycoproteins (MMMGs and HMMGs), which are produced after pathogen infection and modify some physiological functions of the invader (Legaz *et al*., 1998), catalases, which are antioxidant enzymes that detoxify reactive oxygen species (ROS) (Sharma *et al.*, 2012) and WRKY proteins, which comprise a large family of transcription factors that recognize the W box (TTGACC/T) type DNA sequence, which is found in the promoters of many plant defense genes (Rushton *et al.*, 1996).

This review describes defense-related proteins of sugarcane and addresses their putative mechanisms of action, pathogen targets and biotechnological perspectives.

## PR families and their sugarcane homologs

PR proteins were first reported in a tobacco variety hypersensitive to tobacco mosaic virus (TMV) infection (Van Loon and Van Kammen, 1970). PRs are classified into 17 different families, PR-1 to PR-17, based on their primary structure and biological activity (Van Loon *et al.*, 1994; van Loon *et al.*, 2006). New PR families have been proposed, such as the PR-18, which was first purified from sunflower (Custers *et al*., 2004) and the PR-19, which was identified in Scots pine (Sooriyaarachchi *et al*., 2011).

Several sugarcane proteins presenting homology to different PR families were already described. PR proteins in sugarcane are induced by fungi, bacteria, oomycetes, viruses and insects (Table 1). These PR proteins showed antimicrobial or insecticidal activity (Table 1).

**Table 1:** Defense related proteins identified in sugarcane in response to biotic stress.

Protein type	Targets	References
β-1 3-glucanase (PR-2)	Fungi: *Colletotrichum falcatum* and *Sporisorium scitamineum*	Prathima *et al*., 2013; (Su *et al*., 2013)
Chitinases (PR-3, PR-4, PR-8 and PR-11)	Fungi: *Fusarium solani* var. *caeruleum; Fusarium verticillioides; Colletotrichum falcatum; Ceratocystis paradoxa; Gibberella fujikuroi* and *Sporisorium scitamineum*	Franco *et al*., 2014; Medeiros *et al*., 2012; (Que *et al*., 2014)
Thaumatin (PR-5)	Fungi: *Sporisorium scitamineum* and *Colletotrichum falcatum*	Heinze *et al*., 2001; Rocha *et al*., 2007; Sundar *et al*., 2008; (Viswanathan *et al*., 2005)
	Insect: *Diatraea saccharalis*	
Proteinase inhibitors (PR-6)	Fungi: *Trichoderma reesei;*	Ribeiro *et al*., 2008; (Soares-Costa *et al*., 2002)
	Insect: *Sphenophorus levis*	
Peroxidase (PR-9)	Fungi: *Colletotrichum falcatum; Sporisorium scitamineum* and *Puccinia melanocephala*	(Asthir *et al*., 2009)
	Bacteria: *Gluconacetobacter diazotrophicus*	
Ribonuclease-like (PR-10)	Fungi: *Sporisorium scitamineum* and *Puccinia melanocephala*	Oloriz *et al*., 2012; (Que *et al*., 2014)
Defensin (PR-12)	Fungi: *Aspergillus niger; Fusarium solani* and *Neurospora crassa*	(De-Paula *et al*., 2008)
Lipid-transferprotein (PR-14)	Bacteria: *Burkholderia sacchari*	(Borrás-Hidalgo *et al*., 2005)
NBS-LRR protein	Fungi: *Colletotrichum falcatum* and *Puccinia melanocephala*	Borrás-Hidalgo *et al*., 2005; Carmona *et al*., 2004; Glynn *et al*., 2008; Gupta *et al*., 2010; Rossi *et al*., 2003; Selvaraj *et al*., 2014; (Que *et al*., 2009)
	Virus: Sugarcane Yellow Leaf Virus (ScYLV)	
Glycoproteins	Fungi: *Sporisorium scitamineum*	Fontaniella *et al*., 2002; Legaz *et al*., 2011; Martinez *et al*., 1990; Millanes *et al*., 2005; (Millanes *et al*., 2008)
	Bacteria: *Xanthomonas albilineans*	
Catalases	Fungi: *Sporisorium scitamineum; Colletotrichum falcatum* and *Puccinia melanocephala*;	Asthir *et al*., 2009; Kuramae *et al*., 2002; Lambais, 2001; Lao *et al*., 2008; Que *et al*., 2014; Sundar and Vidhyasekaran, 2003; (Su *et al*., 2014b)
	Bacteria: *Gluconacetobacter diazotrophicus* and *Herbaspirilum rubrisubalbicans*	
WRKY proteins	Fungi: *Puccinia melanocephala; Sporisorium scitamineum* and *Colletotrichum falcatum*	Liu *et al*., 2012; Muthiah *et al*., 2013; Prathima *et al*., 2013; Que *et al*., 2014; Santos *et al*., 2015; Sundar *et al*., 2012)

### PR-1 family

The most abundant PR in *Nicotiana tabacum* is in the PR-1 family, with a high level of induction (~10,000-fold) in response to pathogen infection (Alexander *et al*., 1993). PR-1 induction increased the tolerance of different plants, such as tomato, tobacco and Arabidopsis, to pathogens, affecting fungal development (Niderman *et al*, 1995; Segarra *et al*., 2013). In transgenic tobacco plants that overexpress the PR-1a gene, tolerance to infection by two oomycetes, *Peronospora tabacina* and *Phytophthora parasitica* var. *nicotianae*, was improved (Alexander *et al.*, 1993). Proteins in the PR-1 family from tobacco and tomato have different levels of fungicidal activity and affect the germination of *Phytophthora infestans* zoospores (Niderman *et al.*, 1995). PR-1 gene expression was increased in Arabidopsis after the inoculation with *Botrytis cinerea* fungi (Segarra *et al*., 2013).

For sugarcane, a putative PR-1-encoding gene was observed in the SUCEST database (Sugarcane EST Genome Project) (Kuramae *et al.*, 2002). Based on the increased activity of other members of the PR-1 family, showed in tobacco and tomato, against *Phytophthora* species (Alexander *et al.*, 1993; Niderman *et al.*, 1995), PR-1 proteins are relevant targets for future studies against the oomycete species that also affect sugarcane.

### β-1,3-glucanases (PR-2 family)

The PR-2 family includes β-1,3-glucanases, which are enzymes that catalyze the endo-type hydrolytic cleavage of 1,3-β-D-glucosidic linkages in β-1,3-glucans (Leubner-Metzger and Meins Jr, 1999). These enzymes are involved in several physiological and developmental processes in non infected plants (Romero *et al.*, 1998; Leubner-Metzger, 2003; Balasubramanian *et al*., 2012) and in responses to abiotic (Hincha *et al.*, 1997) and biotic factors (Kemp *et al*., 1999; Leubner-Metzger and Meins Jr, 1999). β-1,3-Glucanases release β-glucans from fungal cell walls, which in turn can act as elicitors in plant defense, inducing accumulation of the antibiotic phytoalexin (Sharp *et al.*, 1984; Okinaka *et al*., 1995).

In sugarcane, β-1,3-glucanase genes are differentially expressed after *C. falcatum* (Prathima *et al*., 2013) and *S. scitamineum* (Su *et al.*, 2013) infection, with different expression profiles. In response to both *S. scitamineum* infection and abiotic stresses, *ScGluA1* (KC848050) was up-regulated, whereas *ScGluD1* (KC848051) was slightly down-regulated (Su *et al.*, 2013). The activity was variable for β-1,3-glucanase in genotypes with different levels of susceptibility to *S. scitamineum*. For example, following infection with *S. scitamineum*, glucanase activity increases more rapidly and last longer in a variety of sugarcane resistant to smut than in a susceptible one (Su *et al*., 2013).

### Chitinases (PR-3, PR-4, PR-8 and PR-11 families)

Chitinases are enzymes that hydrolyze the β-1,4-linkage between *N*-acetylglucosamine residues of chitin, which is a structural polysaccharide that is the primary component of cell walls of several types of fungi and exoskeletons of invertebrates (Datta *et al.*, 1999). In the molecular defense of plants, chitinases degrade the chitin in fungal cell walls, with a consequent inhibition of pathogen growth (Schlumbaum *et al.*, 1986). These proteins are grouped in several classes based on sequence similarity and are distributed in four PR families: PR-3, PR-4, PR-8 and PR-11 (Neuhaus, 1999).

Chitinase proteins in sugarcane are associated with responses to biotic and abiotic stresses (Su *et al*., 2014a, 2015). The *ScChiVII1* gene showed differential expression pattern in sugarcane genotypes resistant and susceptible to smut (Wang *et al.*, 2014). Transcript levels of chitinase genes were differentially expressed after infection by the fungi *C. falcatum* (Sundar *et al*., 2008; Rahul *et al.*, 2013), *S. scitamineum* (Su *et al.*, 2014a, 2015; Que *et al.*, 2014) and *Giberella fujikuroi* (Lin *et al.*, 2010). Additionally, the constitutive chitinase activity was higher in sugarcane varieties resistant to red rot disease than in susceptible genotypes (Viswanathan, 2012).

The sugarcane chitinase ScChi, an acidic class III chitinase (PR-8 family), has antifungal activity and inhibits the hyphal growth of *Fusarium solani* var. *coeruleum* (Que *et al.*, 2014). Moreover, sugarcane chitinases showed action against *C. falcatum* and are also associated with *Pseudomonas*-mediated induced resistance (Viswanathan *et al.*, 2003) and with sugarcane response to *D. saccharalis* attack (Medeiros *et al*., 2012).

Sugarcane has two homologs (SUGARWIN1 and SUGARWIN2) of the antifungal barley wound-inducible protein BARWIN, a class II chitinase (PR-4 family) (Medeiros *et al.*, 2012). BARWIN is a basic protein composed of 125 residues, with the tridimensional structure stabilized by three disulfide bonds (Ludvigsen and Poulsen, 1992; Svensson *et al*., 1992). Proteins with a domain similar to BARWIN are observed in several different plants, with (Broekaert *et al*., 1990) or without an associated chitin-binding domain (Friedrich *et al*., 1991; Linthorst *et al.*, 1991; Caruso *et al*., 1999), including *Hevea brasiliensis, Solanum lycopersicum, Nicotiana tabacum* and *Triticum aestivum*. These proteins have antimicrobial activities toward fungi (Hejgaard *et al*., 1992; Caruso *et al*., 1999; Zhu *et al*., 2006) or both fungi and bacteria (Kiba *et al*., 2003).

Transcript levels of genes encoding SUGARWINs were up-regulated in response to mechanical wounding, sugarcane borer (*D. saccharalis*) attack and methyl jasmonate treatment (Medeiros *et al*., 2012). Although induced by *D. saccharalis* damage, SUGARWIN2 proteins have no insecticidal activity; however, these proteins have an antimicrobial role against the opportunistic fungi *Fusarium verticillioides* (Medeiros *et al.*, 2012) and *C. falcatum* (Franco *et al*., 2014), which typically develop after sugarcane borer attacks. Based on these results, SUGARWIN2 proteins are likely involved in a finely regulated defense mechanism in which insect damage induces plant defenses against imminent opportunistic fungi (Medeiros *et al*., 2012; Franco *et al*., 2014). Moreover, the sugarcane pathogenic fungus *C. paradoxa* was affected by SUGARWIN2, but the nonpathogenic fungi *Aspergillus nidulans* and *Saccharomyces cerevisiae* are not (Franco *et al*., 2014). With SUGARWIN2, the morphogenesis and viability of the target fungus were affected by increasing vacuolization, points of fractures and overflow of intracellular material, which lead to cell death (Medeiros *et al.*, 2012; Franco *et al.*, 2014).

### Thaumatin-like proteins (PR-5 family)

Thaumatin-like proteins (TLPs) have a sequence similar to that of thaumatin, a protein extracted from *Thaumatococcus daniellii* (a west African shrub). Thaumatin is a monomeric protein composed of 207 residues and stabilized by eight disulfide bonds (Kim *et al.*, 1988). TLPs are induced by biotic and abiotic stresses (Velazhahan *et al.*, 1999; Rajam *et al.*, 2007). In *in vitro* assays, the plasma membranes (PL) of fungi were disrupted by the antifungal activity of TLPs (Vigers *et al.*, 1992). The mechanisms responsible for the fungal plasma membrane rupture are the direct insertion of TLP in its membrane (formation of pores), causing changes in membrane permeability (Roberts and Selitrennikoff, 1990), or by the hydrolysis of β-1,3-glucans from fungal cell walls (Grenier *et al*., 1999).

TLP was induced in sugarcane after *S. scitamineum* (smut) inoculation (Heinze *et al*., 2001), *C. falcatum* glycoprotein elicitor treatment (Sundar *et al.*, 2008) and in response to *D. saccharalis* attack (Rocha *et al*., 2007). PR-5 genes were differentially expressed after sugarcane challenge with *C. falcatum* (Sathyabhama *et al*., 2015).

### Peptidase inhibitors (PR-6 family)

Peptidase inhibitors (PIs) in plants are important in the control of endogenous and exogenous peptidase activity. The activity of PIs in plant defense is primarily to inhibit the peptidases secreted by insects and pathogenic microorganisms with the digestion of these proteins (Habib and Fazili, 2007).

Plant cystatins, or phytocystatins (PhyCys), are one of the most studied plant protease inhibitors (Benchabane *et al*., 2010). They are competitive and reversible inhibitors of cysteine proteases (Martínez and Díaz, 2008). The genes of the cystatin family have been identified and characterized in some plant species, demonstrating functions in defense against pathogens (Bobek and Levine, 1992; Gutierrez-Campos *et al.*, 1999; Belenghi *et al*., 2003), in response to insect attack (Goulet *et al.*, 2008; Konrad *et al.*, 2008; Liang *et al.*, 2015), in programmed cell death (Solomon *et al.*, 1999; Zhao *et al.*, 2013), in seed germination (Hwang *et al.*, 2009, Zhao *et al.*, 2014), and in responses to abiotic environmental stresses (Hwang *et al.*, 2010). Furthermore, the genes in the cystatin family were differentially expressed in response to different abiotic/biotic stresses, with essential roles in plant defense and hypersensitive cell death (Koiwa*et al*. 2000; Belenghi *et al*. 2003; van der Linde *et al.*, 2012, Wang *et al.*, 2015).

Canecystatin, a sugarcane phytocystatin, is composed of 106 amino acid residues and typically seems to occur as a domain-swapped dimer in solution (Valadares *et al.*, 2013). The canecystatin dimerization mechanism turns this inhibitor inactive, avoiding the inhibition of nontarget endogenous cysteine peptidases (Valadares *et al.*, 2013).

The Sugarcane Genome Project SUCEST was the first to characterize canecystatin (Soares-Costa *et al.*, 2002). Following recombinant expression and purification, the antifungal activity of canecystatin was demonstrated against *Trichoderma reesei* by reducing germination of the filamentous fungus (Soares-Costa *et al.*, 2002). The canecystatin provided inhibitory effect against thiol peptidases, showing that may provide protection for sugarcane against fungi and insects (Oliva *et al.*, 2004). Purified from transgenic sugarcane, His-tagged CaneCPI-1 affects the catalytic activity of cysteine peptidases partially purified from the midgut of the coleopteran *S. levis* (sugarcane weevil; Ribeiro *et al.*, 2008). Furthermore, some sugarcane cystatins present high homology with the *mir 1* gene from maize, which inhibits the growth of a wide range of lepidopteran species (Pechan *et al*., 2000). These results corroborate that cystatins have potential roles in the defense of sugarcane against insect pests.

In addition to the well-described cysteine peptidase inhibitors, sugarcane also has serine peptidase inhibitors of the Bowman-Birk type (BBI) (Mello *et al*., 2003). BBIs are small double-headed serine peptidase inhibitors that are highly stabilized by several disulfide bonds (Birk, 1985). Sugarcane likely has at least 14 BBI genes, with highly variable compositions of the amino acid sequences (Mello *et al*., 2003). The introduction of soybean Bowman-Birk and Kunitz-type serine peptidase inhibitors into sugarcane transgenic lines significantly retards the development of *D. saccharalis*, although the damage caused by this herbivore was not prevented (Falco and Silva-Filho, 2003).

### Endoproteinases (PR-7 family)

The proteins in the PR-7 family are similar to potato alkaline endoproteinase p-69, which is the primary PR in tomato involved in the response to citrus exocortis viroid (CEV) infection (Vera and Conejero, 1988), and to the subtilisin serine protease family (Tornero *et al.*, 1997). Endoproteinases are essential in hydrolyzing peptide bonds in the process of protein degradation. The role of these proteins in biotic defense is unclear, but these proteins may contribute to the dissolution of microbial cell walls (van Loon *et al.*, 2006) or to the posttranslational modification of proteins involved in plant defense (Tornero *et al.*, 1996). The genes *P69B* and *P69C* from tomato inserted into transgenic *Arabidopsis* are induced by salicylic acid and by plant interaction with *Pseudomonas syringae* (Jordá and Vera, 2000).

Endoproteinase genes with diverse substrate specificity are reported in sugarcane, but their involvement in plant defense remains to be explored (Correa *et al.*, 2001; Ramos and Selistre-de-Araujo, 2001; Santos-Silva *et al*., 2012).

### Peroxidases (PR-9 family)

Peroxidases are glycoproteins that catalyze the oxidation of several organic and inorganic substrates by H_2_O_2_ and are involved in a wide variety of physiological and plant defense processes (Chittoor *et al*., 1999). Peroxidases respond to biotic stress by affecting cell wall cross-linking and by creating an unfavorable environment for pathogen growth in plants with the generation of reactive oxygen species (ROS) (Passardi *et al*., 2005). The role of peroxidases in plant cell walls is associated with the biosynthesis of lignin, which is a phenolic biopolymer synthesized for mechanical support and in response to pathogen attack in vascular plants (Østergaard *et al*., 2000). For example, ATP A2 peroxidase in *Arabidopsis thaliana* is a lignin-associated peroxidase that was potentially used in defense against pathogens (Østergaard *et al*., 2000).

In sugarcane, peroxidase activity increases after inoculation with the pathogen *C. falcatum* (red rot) (Sundar *et al*., 2006), with a greater increase in activity in a resistant genotype than in a susceptible one (Asthir *et al*., 2009). Additionally, an elicitor isolated from *C. falcatum* induces peroxidase activity in sugarcane leaves and in suspension-cultured cells (Sundar *et al*., 2002). For sugarcane varieties with different levels of susceptibility to *S. scitamineum*, enzyme activity levels were also variable, with higher increases in activity in a resistant genotype than in a susceptible one after inoculation with the pathogen (Esh *et al*., 2014). Transcripts encoding peroxidase genes were also induced in sugarcane tissues infected by *Gluconacetobacter diazotrophicus* (Lambais, 2001) and during interaction with *Puccinia melanocephala* (Carmona *et al.*, 2004).

### Ribonuclease-like proteins (PR-10 family)

The PR-10 family includes intracellular proteins with ribonuclease activity (Van Loon *et al*., 1994). PR-10 proteins are induced by pathogens in several plants and shown to possess antifungal, antibacterial, antiviral and antinematode activity (Fernandes *et al.*, 2013; Park *et al*., 2004; McGee *et al.*, 2001).

In sugarcane, PR-10 homologs were induced after treatment with the defense-regulator methyl jasmonate (MJ) (Bower *et al*., 2005) and inoculation of sugarcane buds with *S. scitamineum* (Que *et al*., 2014) and in response to *P. melanocephala* infection (Oloriz *et al*. 2012).

### Defensins (PR-12 family)

Plant defensins are small, cysteine-rich antimicrobial peptides found in several organisms, typically with a characteristic β-fold (Stotz *et al*., 2009). These cationic peptides, likely act as antimicrobial molecules that induce the formation of pores in pathogen membranes or modify membrane permeability by a mechanism based on electrostatic charge (Thomma *et al*., 2002).

Sugarcane has at least three putative functional defensins: Sd1, Sd3 and Sd5 (De-Paula *et al*., 2008). Sd1, Sd3 and Sd5 have antifungal activity against the fungi *Aspergillus niger, F. solani* and *Neurospora crassa*; however, these proteins have no antibacterial activity against *Kocuria rhizophila, Bacillus subtilis, Escherichia coli* and *Staphylococcus aureus* (De-Paula *et al*., 2008).

### Lipid-transferproteins (PR-14 family)

Lipid-transfer proteins (LTPs) are small, basic and cysteine-rich lipid-binding proteins in plants that transport lipids between membranes *in vitro* (Rueckert and Schmidt, 1990). These proteins are observed in plant cell walls, with putative roles in cutin biosynthesis and in response to biotic and abiotic stresses (Kader, 1997). Genes encoding the PR-14 type-member, barley LTP4, are differentially induced after fungal and bacterial inoculation (Molina and García-Olmedo, 1993; Molina *et al*., 1996).

After pathogen inoculation, putative homologs of LTPs are differentially induced in a sugarcane genotype resistant to eyespot (*Biopolaris sacchari*; Borrás-Hidalgo *et al*., 2005).

## Other defense-related proteins in sugarcane

### NBS-LRR proteins

Plant NBS-LRR proteins are used to recognize a wide variety of pathogens and insects (Li *et al*. 2015). These proteins encoded by plant resistance genes contain two typical domains: a nucleotide binding site (NBS) and a leucine-rich repeat (LRR). Plant NBS-LRR proteins detect the effector molecules of pathogens that are responsible for virulence. The NBS-LRR class of R genes is categorized into TIR and non-TIR classes based on sequence similarity in the region that precedes the NBS domain. The plant NBS-LRR proteins in the TIR class transport the TOLL/interleukin-1 receptor (TIR) and are called TNL proteins (Joshi and Navak, 2011). The TIR class was found in most dicots but is rare or absent in monocots (Bai *et al.*, 2002; Meyers *et al.*, 2003). The proteins in the non-TIR class are typically called CNL proteins, with most members containing a coiled-coil (CC) N-terminal domain or zinc finger and RPW8 domains (Meyers *et al.*, 2002, DeYoung and Innes, 2006). The CNL class was found in both dicots and monocots (Pan *et al.*, 2000).

Red-rot-related NBS-LRR genes were found in sugarcane EST databases (Gupta *et al.*, 2010); these genes were up-regulated in response to *C. falcatum* challenge, suggesting a possible role in systemic acquired resistance, SAR (Selvaraj *et al*., 2014). These genes were also induced in sugarcane somaclonal variants during interaction with *P. melanocephala* (Carmona *et al.*, 2004). An NBS-LRR class resistance-gene, non-TIR-NBS-LRR-type, was induced in sugarcane in response to infection by *S. scitamineum*, the causal agent of smut (Borrás-Hidalgo *et al.*, 2005;Que *et al.*, 2009).

Resistance-associated genes that encode an NBS domain have been identified in plants using disease resistance gene analog (RGA) markers (Sekhwal *et al*., 2015). RGA sequences are a large set of potential resistance-associated genes with conserved domains. The NBS domain was used to amplify RGA fragments from various plant species (Wang *et al.*, 2001). For example, NBS-RGA analogs from wheat and soybean were used to amplify NBS-LRR DNA in sugarcane; the genes identified were associated with resistance against yellow leaf virus (SCYLV) and moderate resistance against rust caused by *P. melanocephala* (Glynn *et al.*, 2008). Non-TIR-NBS-LRR resistance genes (*Xa1* and *RPS2*) and TIR-NBS-LRR resistance genes (*L6* and *N*) were also identified in sequences of RGAs from smut-resistant sugarcane (Que *et al.*, 2009). Eighteen other sugarcane NBS-LRR gene homologs were found in the SUCEST database with homology to maize and rice varieties resistant to rust (Rossi *et al.*, 2003). Analyses of these genes may enhance the understanding of stress-responsive pathways in sugarcane and lead to the development of markers for disease.

### Glycoproteins

The production of glycoproteins is likely the primary response of sugarcane to infection by pathogens (Fontaniella *et al*., 2002). These macromolecules are found in plant cell walls (Martínez *et al*. 1990) and are of two types, mid molecular mass glycoproteins (MMMGs) or high molecular mass glycoproteins (HMMGs) (Legaz *et al*., 1998).

In response to the entry of a pathogen, sugarcane glycoproteins (MMMGs and HMMGs) are produced that modify some physiological functions of the invader. These glycoproteins were first isolated from sugarcane juice produced in response to mechanical injuries (Legaz *et al*., 1998).

The inoculation of sugarcane with smut teliospores of *S. scitamineum* induces a significant increase in concentration of HMMGs, the polymers from which MMMGs are derived (Martínez *et al*. 1990). In other studies on smut disease, both types of glycoproteins act against the fungus by increasing cytoagglutination and decreasing the germination of teliospores by 50% (Fontaniella *et al*., 2002) or by preventing cell polarization with inhibition of germination tube protrusion and spore germination (Millanes *et al*., 2005). Furthermore, HMMGs and MMMGs produced by healthy sugarcane cause a complete inhibition of smut mycelium growth (Millanes *et al*., 2008).

*Xanthomonas albilineans* is the causal agent of leaf scald, a bacterial-vascular disease in sugarcane. Sugarcane HMMGs and MMMGs act as cell-to-bacterial signals inducing the production of xanthan, an exocellular polysaccharide, by *X. albilineans*. The production of xanthan is likely caused by inhibition of bacterial proteases by these glycoproteins, which consequently protects the enzymes responsible for xanthan biosynthesis from proteolytic degradation (Legaz *et al*., 2011).

### Catalases

Catalase was the first antioxidant enzyme discovered and characterized. In plants, these enzymes detoxify reactive oxygen species (ROS). Catalases are hemeproteins that have high specificity for H_2_O_2_ and catalyze the dismutation of two molecules of H_2_O_2_ into water and oxygen (Sharma *et al.*, 2012).

A search in the SUCEST database found catalases with similarities to the three maize isoforms (CAT 1, CAT 2 and CAT 3) (Soares Netto, 2001). The level of gene expression for a catalase isoform (CAT3) increases after infection with *G. diazotrophicus* (2.5-fold), *Herbaspirillum rubrisubalbicans* (5-fold) (Lambais, 2001) and *S. scitamineum* (Lao *et al.*, 2008). CAT 1 and CAT 3 were also found in sugarcane leaves inoculated with the pathogen *P. melanocephala*, the causal agent of sugarcane rust disease (Kuramae *et al*., 2002).

The elicitor of *C. falcatum* induces variable levels of catalase in suspension-cultured sugarcane cells (Sundar and Vidhyasekaran, 2003). Moreover, high catalase activity was found in two cultivars with varying sensitivity to *C. falcatum* after inoculation with conidia of red rot fungus (Asthir *et al*., 2009).

After inoculation with *S. scitamineum*, the expression of the catalase gene (*ScCAT1*) in sugarcane increases significantly, which suggests that *ScCAT1* protects plants against reactive oxidant-related fungal stimuli. Based on this study, a positive correlation between activity of catalase and smut resistance in sugarcane was also confirmed (Su *et al.*, 2014b). Furthermore, the transcription and expression of the catalase gene were also induced by this interaction (Que *et al.*, 2014).

### WRKY proteins

WRKY proteins are a large family of transcription factors. These proteins are named because of a highly conserved 60 amino acid-long WRKY domain, which is composed of the highly conserved motif WRKYGQK at the N-terminus and a novel metal-chelating zinc finger signature at the C-terminus (Agarwal *et al.*, 2011). WRKY proteins recognize the W box (TTGACC/T) type DNA sequence, which was found in the promoters of many plant defense genes (Rushton *et al.*, 1996). WRKY proteins are involved in differential responses to biotic stresses in plants, either as transcriptional activators or as repressors of pathogen-induced defense programs (Eulgem, *et al.*, 2000; Dong *et al.*, 2003; Ulker and Somssich, 2004; Journot-Catalino *et al*., 2006). Plant WRKY transcriptional factors are activated as part of the plant innate immune system and are triggered by pathogen-associated molecular patterns (PAMP-triggered immunity or PTI) and pathogen virulent effectors (effector-triggered immunity or ETI) (Jones and Dangl, 2006). The high percentage of genes in this family compared with that of other multigene families that also encode plant transcription factors suggests that biotic stresses may have played a key role in the expansion of the WRKY family (Ulker and Somssich, 2004; Agarwal *et al.*, 2011).

Data analyses of sugarcane defense-related genes from many projects worldwide identified WRKY genes (Lambais, 2001; Liu *et al.*, 2012; Wanderley-Nogueira *et al*., 2012; Que *et al.*, 2014; Santos *et al.*, 2015). The expression of β-1,3-glucanases, chitinases, peroxidases and catalases was co-regulated with WRKY-like genes in sugarcane (Dellagi *et al*., 2000; Hara *et al.*, 2000). Furthermore, specific isoforms of sugarcane WRKY-like transcription factor are associated with PR regulons (Lambais, 2001). The expression analysis of WRKY genes indicates strong inductions after sugarcane interaction with *U. scitaminea* (Liu *et al.*, 2012), *C. falcatum* (Sundar *et al*., 2012; Muthiah *et al*., 2013; Prathima *et al*., 2013), *S. scitamineum* (Que *et al.*, 2014) and *P. melanocephala* (Santos *et al.*, 2015).

## Biotechnology potential of defense-related proteins in sugarcane

Induced systemic resistance (ISR) is one strategy that has been described as a potential weapon to improve sugarcane resistance to biotic stresses. Rhizobacterial strains of *Pseudomonas* (nonpathogenic bacteria) were associated with increased resistance in sugarcane to *C. falcatum* by inducing PR proteins in stalk tissues (Viswanathan *et al.*, 2003) and by increasing chitinases with antifungal activity (Viswanathan and Samiyappan, 2001; Viswanathan *et al.*, 2005).

PR proteins from the families PR-13, PR-15, PR-16, PR-17, PR-18 and PR-19, with homologs not yet found in sugarcane, can also be potential targets for RGA markers for enhanced resistance to pathogens. PR-13 (thionins) destroy fungal and bacterial membranes (Bohlmann and Broekaert, 1994) and in barley and *Arabidopsis* inhibited the growth of the sugarcane phytopathogenic fungi *Thielaviopsis paradoxa* (Reimann-Philipp *et al*., 1989) and *Fusarium oxysporum* (Epple *et al*., 1997), respectively. Oxalate oxidases (PR-15 family) and oxalate-oxidase-like proteins (PR-16 family) are correlated with the generation of hydrogen peroxide, which produces a toxic environment to the pathogen or stimulates directly or indirectly plant defense responses (Van Loon *et al*., 2006). Members of the newly described PR-17 family (Nt PRp27 like) were observed in response to *Blumeria graminis* in barley (Christensen *et al.*, 2002), induced by the synthetic benzo (1,2,3) thiadiazole-7-carbothioic acid S-methyl ester (BTH) in wheat (Görlach *et al*., 1996) and upon mosaic virus infection in tobacco. PR-18 (fungus- and SA-inducible carbohydrate oxydases) enhanced resistance to infection by bacteria in tobacco transgenic plants (Custers *et al*., 2004). Recently, a new PR protein with antimicrobial effect was identified in *Pinus sylvestris* and named PR-19 (Sooriyaarachchi *et al*., 2011). This protein binds to fungal cell wall glucans altering cell wall structure which leads to morphological distortion of hyphae (Sooriyaarachchi *et al*., 2011).

Recently, RNA interference also has been used for control of sugarcane diseases. RNAi of endochitinases in the sugarcane endophyte *Trichoderma virens* 223 was used as a form of biocontrol for *C. paradoxa* (Romão-Dumaresq *et al.*, 2012). RNAi has been used for development of viral resistant plants (Kim *et al.*, 2013; Ntui *et al*., 2013). Gene silencing has proven to be effective to obtain multistrain resistant sugarcane plants for mosaic disease (Potyvirus sugarcane mosaic virus - ScMV and/or Sorghum mosaic virus - SrMV)(Guo *et al*., 2015).

Studies have reported that microRNA-guided gene regulation was essential for tolerance to biotic stresses (Gupta *et al.*, 2014). Thiebaut *et al*. (2012) identified several microRNAs in sugarcane after inoculation with *Acidovorax avenae* subsp *avenae*. These new microRNAs have a potential use for genetic engineering of stress-resistant plants and can contribute to an improved understanding of regulatory pathways for defense-related proteins.

The development of transgenic plants is another strategy proposed to increase resistance to pathogens. For example, the insertion of single genes encoding PR proteins, such as β-1,3-glucanase (Sundaresha *et al.*, 2010) or thaumatin-like proteins (Chen *et al.*, 1999; Datta *et al.*, 1999; Velazhahan and Muthukrishnan, 2003; Schestibratov and Dolgov, 2005), in transgenic plants increases host resistance against fungi. Additionally, transgenic plants that express more than one PR-encoding gene have also been considered as a strategy to improve plant resistance to pathogens (Anand *et al.*, 2003; Amian *et al.*, 2011).

## Concluding remarks

Sugarcane is one of the most important commodities worldwide, primarily for sugar and biofuel production. However, despite the significant agronomical relevance, little is known about the role of proteins in sugarcane defense when compared with other plant species. Improving our knowledge of sugarcane defense mechanisms against pathogens is still a great challenge to be achieved.
